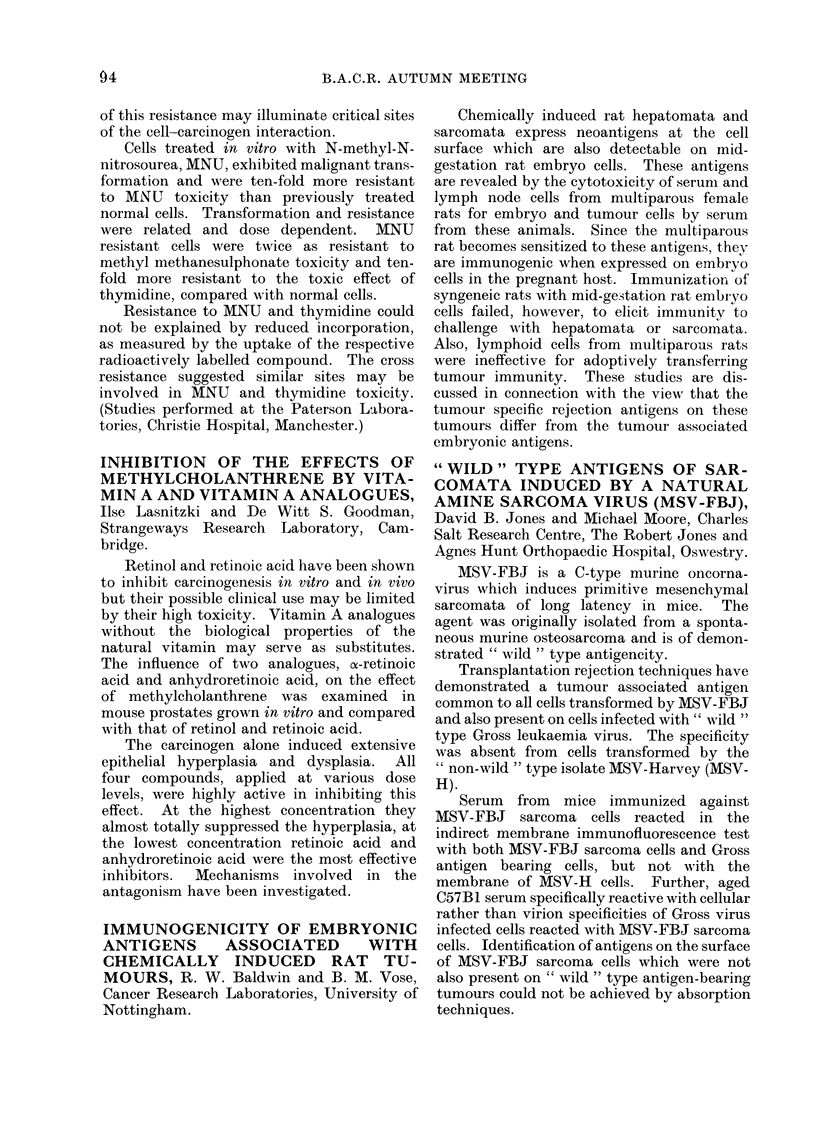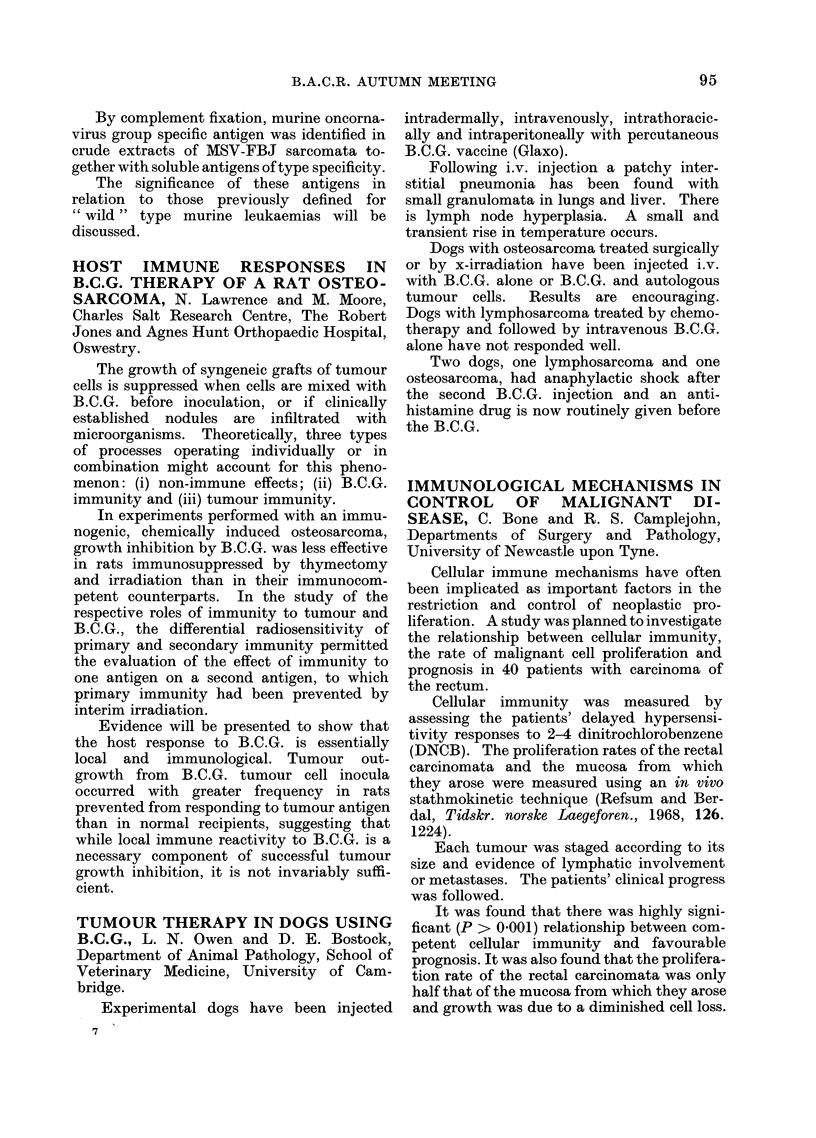# Proceedings: "Wild" type antigens of sarcomata induced by a natural amine sarcoma virus (MSV-FBJ).

**DOI:** 10.1038/bjc.1974.22

**Published:** 1974-01

**Authors:** D. B. Jones, M. Moore


### " WILD " TYPE ANTIGENS OF SARCOMATA INDUCED BY A NATURAL AMINE SARCOMA VIRUS (MSV-FBJ),

David B. Jones and Michael Moore, Charles Salt Research Centre, The Robert Jones and Agnes Hunt Orthopaedic Hospital, Oswestry.

MSV-FBJ is a C-type murine oncornavirus which induces primitive mesenchymal sarcomata of long latency in mice. The agent was originally isolated from a spontaneous murine osteosarcoma and is of demonstrated " wild " type antigencity.

Transplantation rejection techniques have demonstrated a tumour associated antigen common to all cells transformed by MSV-FBJ and also present on cells infected with " wild " type Gross leukaemia virus. The specificity was absent from cells transformed by the " non-wild " type isolate MSV-Harvey (MSV-H).

Serum from mice immunized against MSV-FBJ sarcoma cells reacted in the indirect membrane immunofluorescence test with both MSV-FBJ sarcoma cells and Gross antigen bearing cells, but not with the membrane of MSV-H cells. Further, aged C57B1 serum specifically reactive with cellular rather than virion specificities of Gross virus infected cells reacted with MSV-FBJ sarcoma cells. Identification of antigens on the surface of MSV-FBJ sarcoma cells which were not also present on " wild " type antigen-bearing tumours could not be achieved by absorption techniques.

By complement fixation, murine oncornavirus group specific antigen was identified in crude extracts of MSV-FBJ sarcomata together with soluble antigens of type specificity.

The significance of these antigens in relation to those previously defined for "wild" type murine leukaemias will be discussed.